# Spectroscopic, antioxidant and cytotoxicity studies of vanillic acids

**DOI:** 10.1038/s41598-025-13965-6

**Published:** 2025-08-26

**Authors:** Renata Swislocka, Natalia Kowalczyk, Aleksandra Dabrowska, Renata Choinska, Wlodzimierz Lewandowski, Grzegorz Swiderski

**Affiliations:** 1 Department of Chemistry, Biology and Biotechnology, Bialystok University of Technology, Wiejska 45E, 15-351 Bialystok, Poland; 2 Prof. Waclaw Dabrowski Institute of Agriculture and Food Biotechnology–State Research Institute, Rakowiecka 36, 02-532 Warsaw, Poland; 3 Faculty of Health and Life Sciences, INTI International University, Persiaran Perdana BBN, Putra Nilai, 71800 Nilai, Negeri Sembilan Malaysia

**Keywords:** Vanillic acid, Isovanillic acid, *o*-vanillic acid, Antioxidant activity, Cytotoxicity, Spectroscopic studies, Computational research, Computational biology and bioinformatics, Microbiology, Environmental sciences

## Abstract

Vanillic acid is a phenolic compound recognized for its use as a flavoring agent in the food industry. It can be found in a variety of products, such as thyme, rice, oranges, cherries, green tea, wine, and beer. Vanillic acid has been substantiated to show various beneficial pharmacological properties including anti-inflammatory, antibacterial, cardioprotective, hepatoprotective, antitumorigenic, free radical scavenging, antioxidant and even antivenomous activity. Since vanillic acid has low bioavailability, and bioavailability of a compound depends (among other factors) on its chemical structure, testing isomers of vanillic acid may yield promising results. Several structural and biological studies were carried out to determine the correlation between the molecular structure and biological activity of vanillic acid and its isomers (isovanillic acid and *o*-vanillic acid). Studies have shown that changes in the electronic structure of vanillic acids affect their different reactivity, antioxidant activity, and cytotoxicity. *O*-vanillic acid, characterized by the highest reactivity, is a weak antioxidant and the best cytotoxic compound. No direct correlation was observed between the antioxidant activity of vanillic acids and their toxic effect on model cell lines, probably due to the different mechanisms of action in the phenomena studied.

## Introduction

Vanillic acid (4-hydroxy-3-methoxybenzoic acid, VA)^1^ is an oxidized form of an aldehyde - vanillin^2,3^ and also its principal metabolite^1^. It is mostly known for its use as a flavoring agent in the food industry^2,4^ but also serves as a precursor for synthesizing bio-based polymers and is used in the production of various active pharmaceutical compounds, including brovanexine, vanitiolide, etamivan, and flecainide^3^.

VA is an endogenous metabolite of noradrenaline and adrenaline^1^ and its presence has been detected in the human brain and cerebrospinal fluid^5^. It also naturally occurs in various plants, including herbs: *Angelica sinensis*, *Ocimum basilicum*, *Origanum vulgare*, *Salvia Rosmarinus*, *Thymus vulgaris*; grains (e.g. rice and corn); fruits and vegetables: *Euterpe oleracea*, *Phoenix dactylifera*, *Olea europaea*, oranges, guavas and cherries. Other good sources of VA are green tea^6,7^, beer, and wine^8^.

VA belongs to the group of benzoic acid derivatives^2^, and as a phenolic compound, exhibits diverse therapeutic properties^8^. Numerous scientific studies indicate various pharmacological properties of VA: anti-inflammatory, antibacterial, cardioprotective, hepatoprotective, antitumorigenic, free radical scavenging, antioxidant and even antivenomous (it has been determined that VA selectively and specifically inhibits the anticoagulant effect of some snake venom proteins^1^ activities^3,4,9–12^.

DNA is the main target of free radicals generated in the cell and therefore many current studies on the effects of phenolic compounds are not only concerned with cytotoxicity but also aim to determine the genotoxic effects against oxidative DNA damage. Antioxidant compounds can attenuate the effects of DNA damage. Inhibition of DNA damage and/or enhancement of DNA repair activity by dietary means is an important strategy to prevent mutations and carcinogenesis^13^. Diet rich in vanillic acid could reduce free radical cancer promotion and retard mutagenesis caused by chemical and physical mutagens in different models^4^.

VA insulates the biological membrane and inhibits lipid peroxidation in cells. It can also scavenge and remove free radicals, such as hydroxyl radicals and lipid peroxyl radical^4^. Tai et al. reported that VA showed greater ABTS radical scavenging activity than ascorbic acid and trolox. They also found that it had superior efficacy compared to ascorbic acid and trolox in terms of oxygen radical scavenging capacity and mechanisms of inhibiting oxidative hemolysis^14^. It is also suggested that VA could prevent oxidative damage to DNA and chromosomes when used at an appropriately low dose^15^. Tai et al. also reported that VA and its esters have carboxyl and carboxylate ester groups, which are generally considered to have electron-donating and radical-scavenging capacities depending on the nature of the substituted groups^16^. VA reduces oxidative-related markers such as superoxide dismutase, glutathione, and glutathione peroxidase^11^. As a potent antioxidant and free radical scavenger, VA can prevent premature skin aging caused by free radicals generated by UV exposure. When used topically, VA has strong transdermal penetration capacity into the epidermis and dermis, which makes it a good candidate for use in cosmetics applied to the skin (ointments, lotions)^3^.

Beneficial properties of VA include bioactivity against diabetes and obesity^12^. When diabetic rats were given oral VA, their fasting plasma glucose, insulin, and blood pressure levels were significantly lower than in the diabetic control group. In diabetic hypertensive rats treated with VA, antioxidant activities were significantly improved, and lipid peroxidation markers were markedly reduced. These findings indicate that VA has a modulatory impact on diabetic hypertension regulation by lowering blood glucose, insulin, and blood pressure while also combating oxidative stress via tissue activation^6^. VA is also suggested to be a useful therapeutic candidate for colitis^2^.

Additionally, VA has been shown to have cardioprotective properties^8,12^ thanks to its ability to improve mitochondrial function, scavenge free radicals, and reduce levels of lipid peroxidation^8^. VA has also been demonstrated to block pro-inflammatory cytokines and suppress inflammatory cascades^11^.

While VA has great potential to be used as a nutraceutical and provides scope for therapeutic applications, its oral bioavailability (about 25%) is limited due to its rapid metabolism and poor solubility^12,17^. Since the bioavailability of a compound depends, among other factors, on its chemical structure^18–20^, investigating VA isomers might yield promising results.

**Fig. 1:**
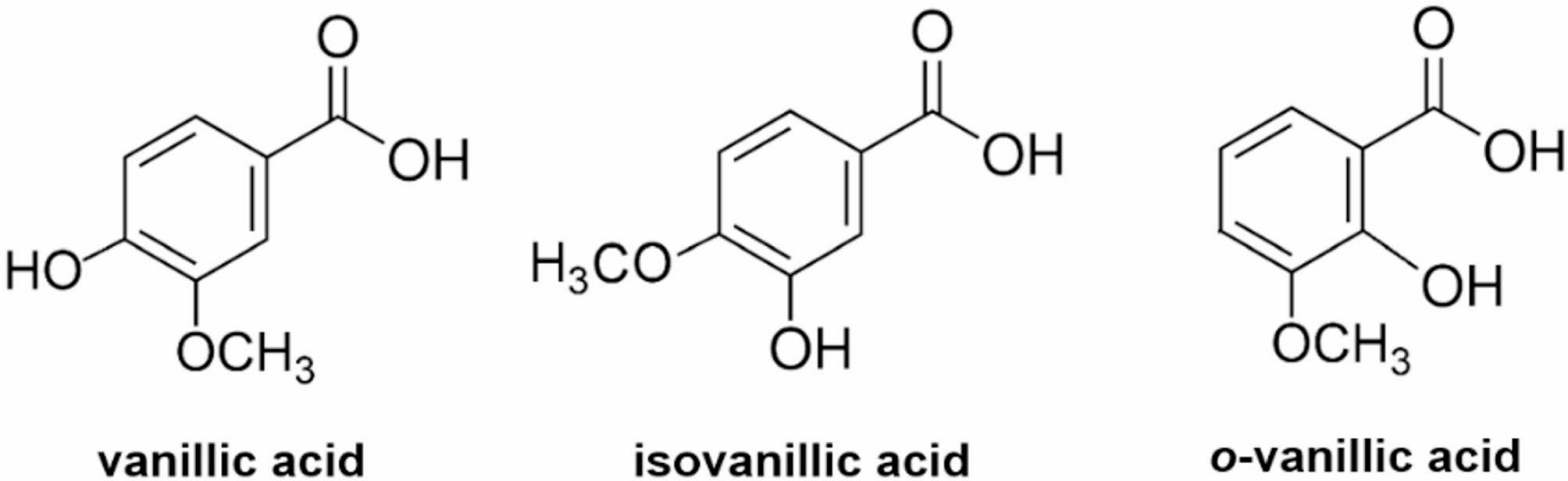
The structural formulas of vanillic acid, isovanillic acid and *o*-vanillic acid.

The purpose of the presented research was to evaluate the correlation between the molecular structure of natural ligands - VA and its isomers (Fig. 1). and their biological activity, especially in terms of antioxidant activity. VA and its isomers differ in the mutual position of hydroxyl and methoxy groups in the aromatic ring relative to the carboxyl group. These structural differences are a potential reason why these chemical compounds exhibit differences in reactivity and biological activity.

To determine the correlation between the molecular structure and biological activity of tested compounds, several structural (using spectroscopic methods: FT-IR, UV-VIS), theoretical calculations in the Gaussian program (theoretical spectroscopic spectra, electronic charge distribution, electronic parameters, energies of HOMO and LUMO orbitals, ESP potential and others), antioxidant, and cytotoxic-assays were carried out.

## Results

### Theoretical calculated

#### Structures and HOMO LUMO orbitals

**Fig. 2:**
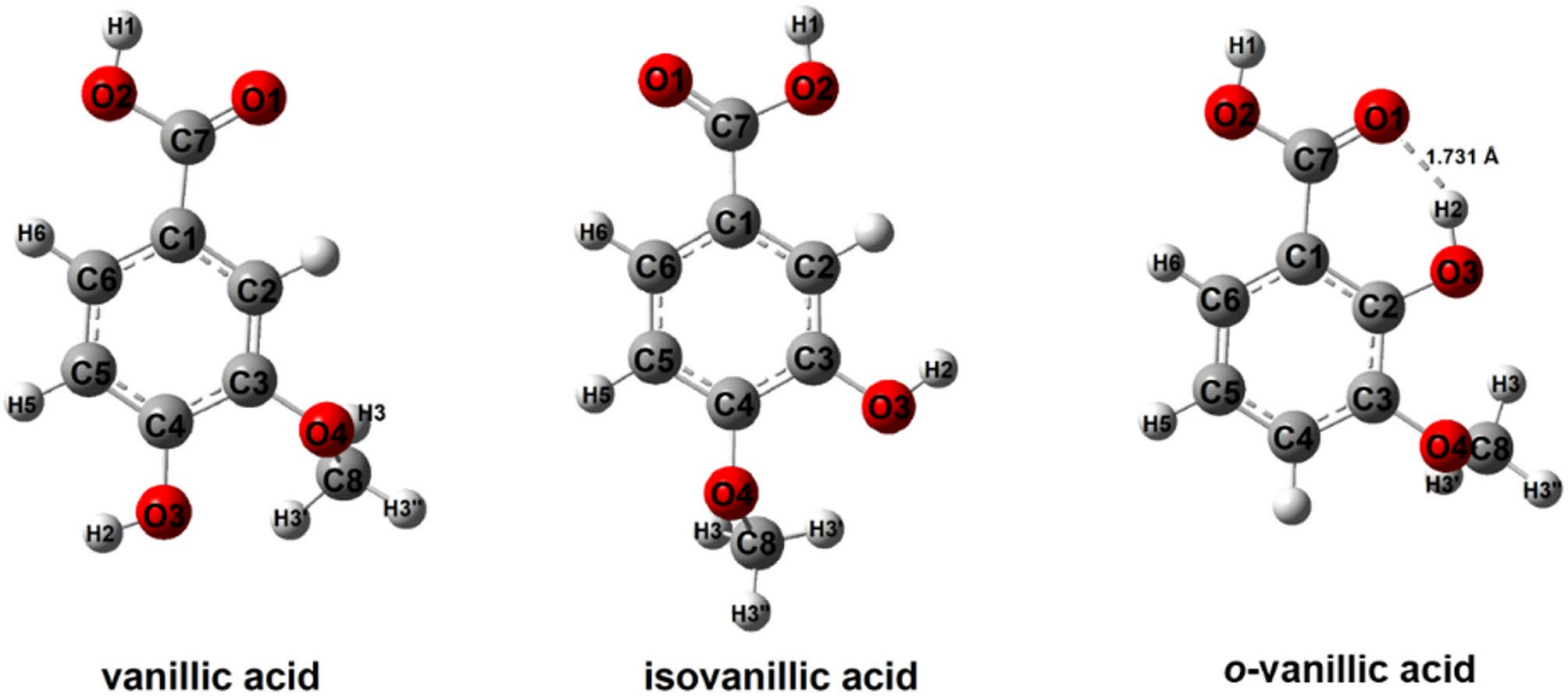
The structures of vanillic, isovanillic and *o*-vanillic acids calculated in B3LYP/6-311G++(d, p).

**Fig. 3:**
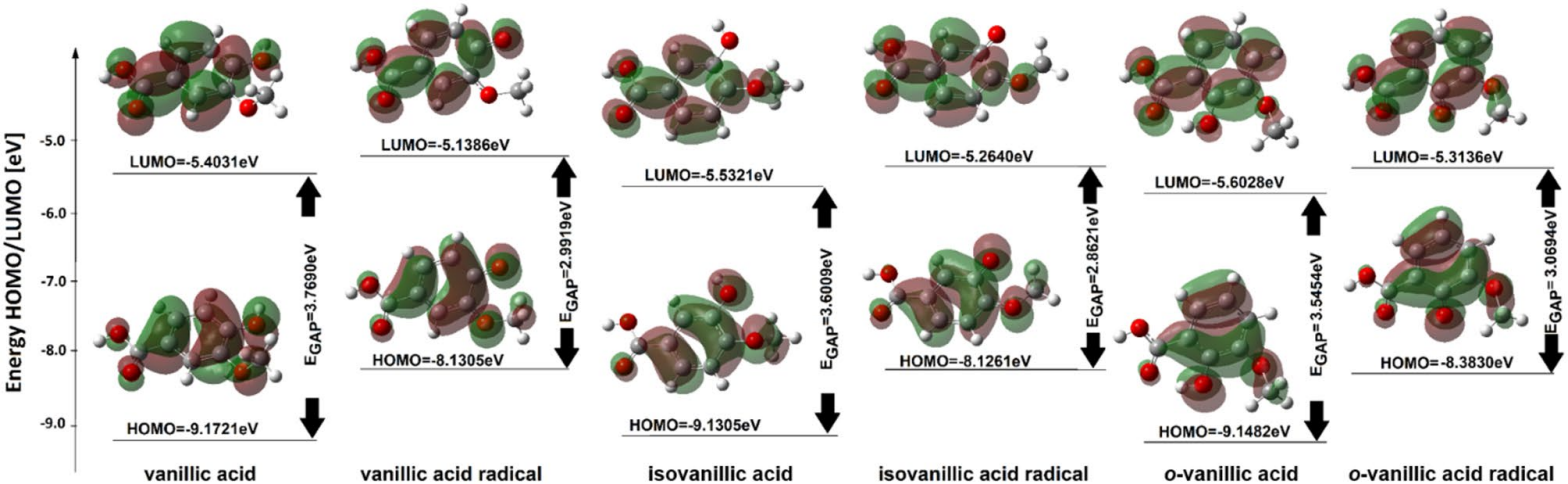
Molecular orbital shapes and their energy values for vanillic acids and their radicals.

Figure 2 presents the modeled structures of vanillic, isovanillic and *o*-vanillic acids. For the optimized structures of neutral molecules and radicals, the energies of frontier orbitals (HOMO and LUMO), reactivity descriptors and geometric aromaticity indices were calculated (Table 1). The shapes of HOMO and LUMO molecular orbitals are shown in Fig. 3.

**Table 1:** Frontier orbitals energy values, reactivity descriptors and geometric aromaticity indices calculated in B3LYP/6-311 + + G(d, p) (water solution – CPCM conductor-like polarizable continuum model) for vanillic, isovanillic and *o*-vanillic acids and their radicals.

	VA	VA radical	isoVA	isoVA radical	o-VA	o-VA radical
Energy [eV]	-16619.4	-16,602	-16619.4	-16,602	-16619.5	-16601.7
Dipole moment [D]	5.5536	0.8054	4.0729	1.8348	3.4011	8.0616
E_HOMO_ [eV]	-9.1721	-8.1305	-9.1330	-8.1261	-9.1482	-8.3830
E_LUMO_ [eV]	-5.4031	-5.1386	-5.5321	-5.2640	-5.6028	-5.3136
Δ = E_LUMO_-E_HOMO_ [eV]	3.7690	2.9919	3.6009	2.8621	3.5454	3.0694
Ionisation potential I=-E_HOMO_	9.1721	8.1305	9.1330	8.1261	9.1482	8.3830
Electron affinity A=-E_LUMO_	5.4031	5.1386	5.5321	5.2640	5.6028	5.3136
Electroegativity Χ=(I + A)/2	7.2876	6.6345	7.3325	6.6951	7.3755	6.6295
Chemical potential µ=-(I + A)/2	-7.2876	-6.6345	-7.3325	-6.6951	-7.3755	-6.8483
Chemical hardness η=(I-A)/2	1.8845	1.4959	1.8004	1.4310	1.7727	1.5347
Chemical softness S = 1/(2η)	0.2653	0.3342	0.2777	0.3494	0.2821	0.3258
Electrophilicity index ω = µ^2^/2η	14.0909	14.7122	14.9313	15.6613	15.3434	15.2793
Aromaticity indices						
HOMA	0.959	0.392	0.966	0.494	0.922	0.181
GEO	0.019	0.392	0.012	0.311	0.037	0.532
EN	0.022	0.216	0.022	0.195	0.041	0.287
I6	92.87	66.98	94.22	70.73	89.88	62.35
BAC	0.874	0.498	0.900	0.572	0.856	0.466
Aj	0.992	0.830	0.994	0.864	0.983	0.770

Based on the geometric data of the optimized structures (bond lengths in the ring), aromaticity indices were calculated. The distribution of the electronic charge in the aromatic ring affects the reactivity of aromatic compounds. A stable aromatic ring with equalized bond lengths is less susceptible to substitution reactions than a ring in which the π-electron system has been disturbed. Substituents substituted in the ring influences the change in the aromatic system of chemical compounds. Hydroxyl and methoxy groups substituted in different positions relative to each other affect the aromatic system in vanillic acids. Geometric aromaticity indices for purely aromatic systems (with balanced bonds) take the value of 1 (HOMA index, I6, Aj, BAC) and for non-aromatic rings − 0. In the case of the Bird Index, the value is 100 - for purely aromatic systems (benzene) and 0 for non-aromatic systems (cyclohexatriene). IsoVA is characterized by the highest aromaticity among the acids tested (Table 1). VA is characterized by slightly lower aromatic ring stability than isoVA. It can be expected that both acids will be characterized by similar reactivity. *O*-VA, in comparison to the other two vanillic acids, shows markedly lower aromaticity. The hydroxyl group in the molecule of this acid is substituted in close proximity to the carboxyl group, as a result of which it is possible to form an intramolecular hydrogen bond between the hydrogen atom of the hydroxyl group and the oxygen atom of the carbonyl group (Fig. 2.). The calculated distance of this bond is 1.731 Å. The diffraction data^21^ indicate that these acids form dimers, and in the case of *o*-VA acid there is also an intramolecular hydrogen bond between the hydrogen atom of the hydroxyl group and the oxygen atom of the carbonyl group. The length of this bond is 1.900 Å. The lengths of the O-H…O hydrogen bonds are 2.607 Å (calculated) and 2.617 (diffraction data), respectively. The presence of this bond increases the disorder disruption of the aromatic system in *o*-VA.

Homolytic cleavage of the hydrogen-oxygen bond in the hydroxyl group of the aromatic ring leads to the formation of a radical. The unpaired electron on the oxygen atom of this group exerts strong interactions on the electronic system of the aromatic ring. The calculated aromaticity indices for VA radicals showed a significant decrease in the aromaticity of the π-electron system after the formation of the radical (Table 1). It was observed that the less stable the aromatic system in the acid molecule, the greater the decrease in aromaticity of this system upon radical formation. The energy gap between the energy of the frontal orbitals HOMO and LUMO (EGAP) is a measure of the reactivity of the molecule^22^. Figure 3 shows the shapes of the HOMO and LUMO orbitals. It was observed that the HOMO orbitals, similarly to LUMO, occupy the region of ​​the atoms of the π-electron system of the modelled molecules. It should therefore be expected that the aromaticity of the π-electron system of the tested molecules will correlate well with the EGAP value and the hardness/softness of the molecules^23^. Among the tested molecules, *o*-VA is characterized by the lowest EGAP energy value, as well as the lowest hardness (the highest softness). Therefore, it will be the molecule most susceptible to reactions. The calculated values of the aromaticity indices (the lowest aromaticity) also indicate that this molecule will be the most susceptible to reactions. Acid radicals are characterized by a lower GAP energy than their corresponding acids, which indicates that they are more reactive than neutral molecules. They are also characterized by higher softness values and lower hardness values than neutral molecules.

#### NBO and electrostatic potential maps

The NBO electron charge distribution was calculated for neutral vanillic acids molecules in the gas phase and the aqueous phase (CPMC model) and for radicals in the aqueous phase (CPMC model). The calculations are summarized in Table 2.

**Table 2:** NBO calculated in B3LYP/6-311 + + G(d, p) (water solution model – CPCM) for vanillic, isovanillic and *o*-vanillic acids and their radicals.

Atom	o-VA			VA			isoVA		
	Gas	Water	Radical	Gas	Water	Radical	Gas	Water	Radial
C1	-0.212	-0.243	-0.135	-0.182	-0.196	-0.111	-0.169	-0.171	-0.182
C2	0.332	0.343	0.254	-0.236	-0.175	-0.214	-0.232	-0.228	-0.159
C3	0.256	0.253	0.331	0.256	0.257	0.356	0.267	0.276	0.327
C4	-0.252	-0.188	-0.203	0.313	0.313	0.360	0.299	0.293	0.372
C5	-0.219	-0.231	-0.125	-0.245	-0.271	-0.222	-0.221	-0.226	-0.261
C6	-0.160	-0.155	-0.150	-0.151	-0.152	-0.179	-0.165	-0.173	-0.106
C7	0.792	0.810	0.805	0.787	0.799	0.790	0.788	0.801	0.800
C8	-0.200	-0.200	-0.192	-0.202	-0.200	-0.197	-0.200	-0.201	-0.196
O1	-0.616	-0.674	-0.598	-0.607	-0.650	-0.629	-0.601	-0.644	-0.632
O2	-0.665	-0.671	-0.722	-0.700	-0.692	-0.682	-0.697	-0.690	-0.684
O3	-0.644	-0.686	-0.421	-0.661	-0.674	-0.575	-0.679	-0.686	-0.570
O4	-0.573	-0.589	-0.579	-0.571	-0.588	-0.490	-0.561	-0.584	-0.496
H1	0.480	0.506	0.507	0.485	0.500	0.505	0.486	0.502	0.504
H2	0.487	0.508	-	0.488	0.495	-	0.473	0.493	-
H3	0.194	0.180	0.173	0.193	0.172	0.196	0.165	0.173	0.195
H3’	0.174	0.172	0.170	0.175	0.180	0.192	0.187	0.184	0.195
H3’’	0.174	0.189	0.191	0.175	0.190	0.196	0.185	0.190	0.192
H5	0.221	0.221	0.229	0.222	0.225	0.225	0.222	0.228	0.233
H6	0.230	0.230	0.238	0.231	0.231	0.236	0.231	0.230	0.234

The ^−^OH and ^−^O^−^CH_3_ substituents in the aromatic ring of the acids studied cause a significant decrease in electron density around the carbon atoms to which they are attached. The lowest electron density around the carbon atoms to which the hydroxyl group is attached was noted for *o*-VA, and the highest for isoVA. The lowest electron density around the carbon atom to which the methoxy group is substituted was noted for isoVA. The electron charge distribution in the aromatic ring is the most disturbed (the greatest differentiation of electron charges in the aromatic ring) in the case of *o*-VA (the lowest aromaticity). Significant changes in the electron charge distribution of the aromatic ring of the radicals were observed with respect to the corresponding molecules of neutral acids. The largest changes in the π-electron charge distribution after the formation of the radical were observed in the case of *o*-VA, and the smallest in the case of isoVA.

**Fig. 4:**
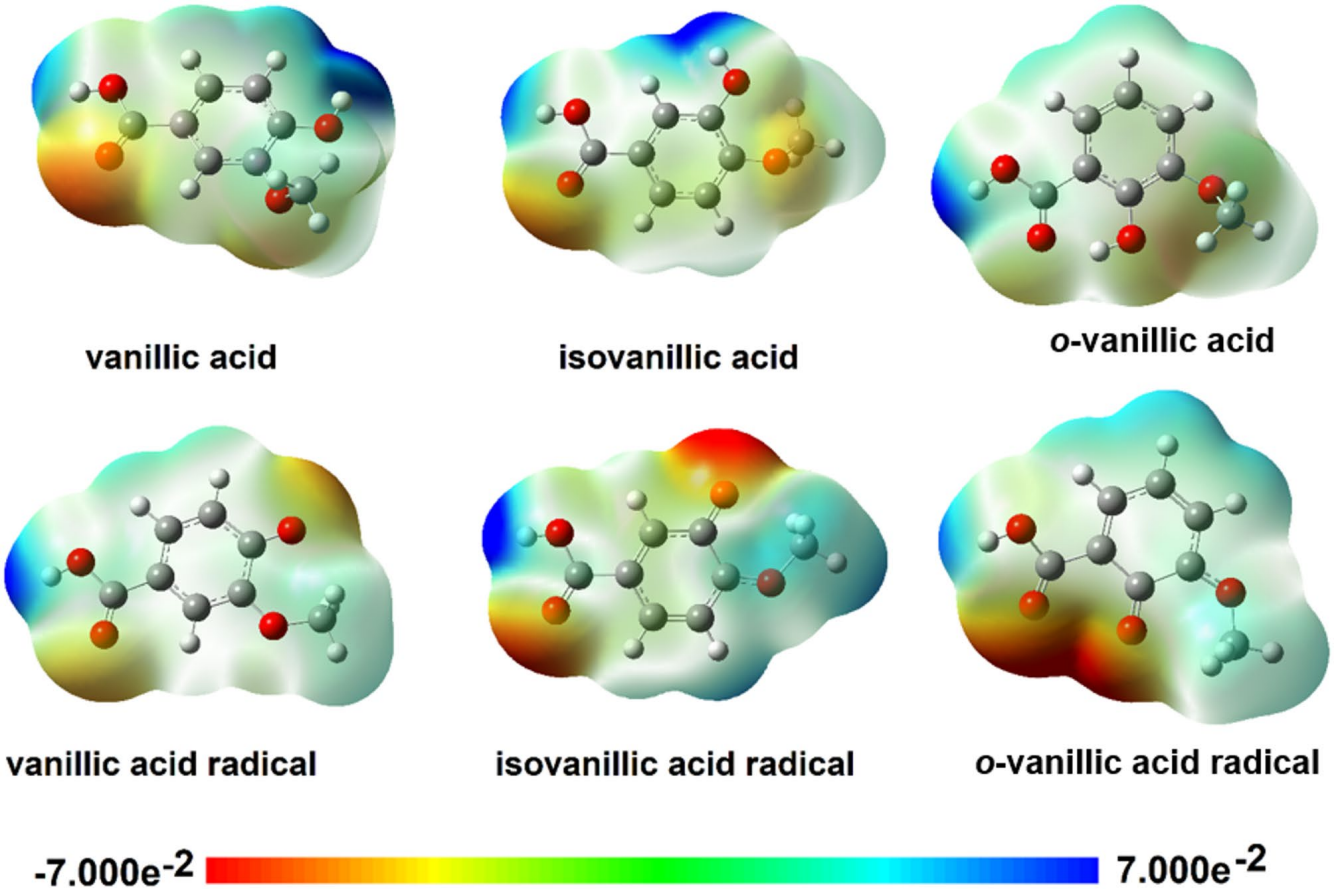
Electrostatic potential maps for vanillic acids and their radicals.

Electrostatic potential maps were modeled using the SCF method for structures optimized by the B3LYP/6-311 + + G(d, p) method for neutral molecules and radicals in an aqueous environment (CPCM) (Fig. 4). The electrostatic potential map displays electrophilic (red) and nucleophilic (blue) regions in the molecules (Fig. 4). In the molecules of VA and isoVA, increased susceptibility to electrophilic reactivity is characterized by the oxygen atom at the carbonyl group and the oxygen atom of the methoxy group. In the *o*-VA molecule, in which there is an intramolecular hydrogen bond between the oxygen atom of the carbonyl group and the hydrogen atom of the hydroxyl group of the aromatic ring, a reduced susceptibility to electrophilic reactions was observed in the carboxyl group. In the *o*-VA molecule, a shift of the electron cloud from the oxygen atom of the carbonyl group towards the hydroxyl group was observed. Areas of nucleophilic activity were identified around the hydroxyl groups of the aromatic ring and the hydroxyl group in the carboxyl group. The formation of the radical is accompanied by a change in the susceptibility of the molecule in the area of the hydroxyl groups from a nucleophilic character to a clearly electrophilic character.

The oxygen atom with an unpaired electron becomes more susceptible to electrophilic reactions. The reactivity of radicals is much greater than the reactivity of neutral molecules. In the case of the *o*-VA radical, a significant increase in the ability of the carbonyl group and the atom with an unpaired electron toward electrophilic interactions is observed. Radicals are molecules that are more reactive than the acids from which they were formed.

### Energy reaction with hydroxyl radical

**Fig. 5:**
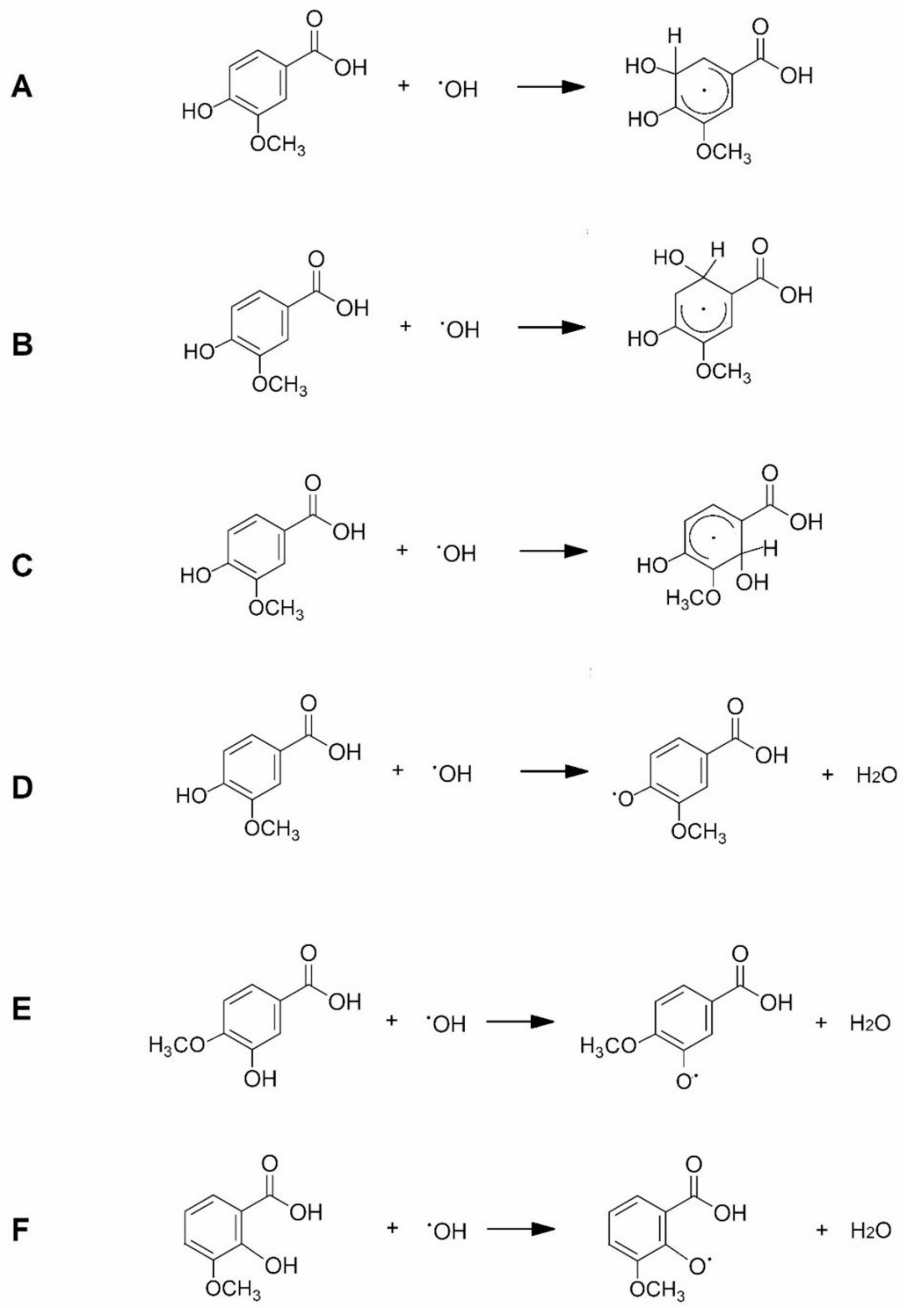
Mechanisms of the reaction of hydroxyl radicals with vanillic acids. (**A**–**C**) attachment of the hydroxyl radical to the aromatic ring of vanillic acid, (**D**) hydrogen transfer (HAT mechanism) in vanillic acid, (**E**) hydrogen transfer (HAT mechanism) in isovanillic acid, (**F**) hydrogen transfer (HAT mechanism) in *o*-vanillic acid.

The energy of the reaction between the hydroxyl radical and the acids in an aqueous environment was calculated. Calculations were performed for reactions occurring according to the HAT mechanism (hydrogen atom transfer) shown in Fig. 5. The structures of the acid and radical molecules were optimized using the B3LYP/6-311 + + G(d, p) method. The reaction energy was calculated based on the difference in energy of the products and substrates. As a result of the reaction of vanillic acids with a hydroxyl radical, a proton of the hydroxyl group of the aromatic ring of the acid is split off and an acid radical molecule and a by-product – a water molecule – are formed (Fig. 5D,E). This reaction proceeds with an intermediate step in which the hydroxyl radical attaches to the aromatic ring of the acid to form a radical – an unpaired electron appears in the aromatic system. The radical can be attached in one of three places on the aromatic ring (Fig. 5A-C). The energy of the intermediate reaction (Fig. 5A-C) was calculated for vanillic acid. Reaction energy calculations showed (Table 3) that the preferred site of attachment of the hydroxyl radical to the vanillic acid molecule is the position closest to the hydroxyl group of the acid. This reaction was characterized by the lowest energy expenditure.

**Table 3:** Eaction energies of vanillic, isovanillic and *o*-vanillic acids with hydroxyl radical. (**A**–**C**) addition of hydroxyl radical to the aromatic ring of vanillic acid, (**D**) hydrogen transfer (HAT mechanism) in vanillic acid, (**E**) hydrogen transfer (HAT mechanism) in isovanillic acid, (**F**) – hydrogen transfer (HAT mechanism) in *o*-vanillic acid.

Reaction (Fig. 5.)	Energy of neutral molecule *10^3^ [kJ/mol]	Hydroxy radical energy [kJ/mol]	Radical energy *10^3^ [kJ/mol]	Water energy [kJ/mol]	Reaction energy [kJ/mol]
**A**	-1603.552	-196.302	-1802.542	-200.763	2687.75
**B**	-1603.552		-1802.547		2692.59
**C**	-1603.552		-1802.553		2698.78
**D**	-1603.552		-1601.872		2780.88
**E**	-1603.548		-1601.873		2786.19
**F**	-1603.561		-1601.843		2742.04

The energy of these reactions was calculated based on the energy difference:

$${{\text{E}}_{{\text{reaction}}}}={{\text{E}}_{{\text{radical}}}} - \left( {{{\text{E}}_{{\text{neutral}}}} - {\text{E}}_{{{\text{OH}}}}^{ \cdot }} \right),$$

where: E_reaction_- reaction energy, E_radical_- Energy of the radical formed, E._OH_- energy of the hydroxyradical.

The energy of the reaction of the hydroxyl radical with vanillic, isovanillic and *o*-vanillic acids was also calculated (Fig. 5D–F) (Table 3). The reaction energy was calculated according to the scheme:

$${{\text{E}}_{{\text{reaction}}}}=\left( {{{\text{E}}_{{\text{radical}}}}+{{\text{E}}_{{{\text{H}}_2}{\text{O}}}}} \right) - \left( {{{\text{E}}_{{\text{neutral}}}}+{\text{E}}_{{{\text{OH}}}}^{ \cdot }} \right),$$

where: E_reaction_- reaction energy, E_radical_- Energy of the radical formed, E._OH_- energy of the hydroxyradical, E_H2O_- energy of water molecule.

Calculations have shown that the lowest reaction energy is characteristic of the reaction of *o*-VA with the hydroxyl radical, while the highest energy is required for the reaction of the radical with isoVA. The different position of the hydroxyl group relative to the methoxy group in the aromatic ring of vanillic acids influences the antioxidant capacity of these compounds. Calculations of the stabilization of the π-electron system (aromaticity indices) and the energies of frontal orbitals and reactivity descriptors (EGAP, softness, chemical hardness) indicate that *o*-VA is the most reactive compound, which is confirmed by calculations of the reaction energy of acids with the hydroxyl radical. IsoVA is a molecule with the highest aromaticity - the most stable molecule, which is why the reaction energy of this molecule with the hydroxyl radical is higher. In our current research we used and relied mainly on the HAT model and theory, but we have not fully explored the SPLET (sequential proton loss electron transfer) and SET (single electron transfer) mechanisms due to the additional complexity of their modeling.

**Fig. 6:**
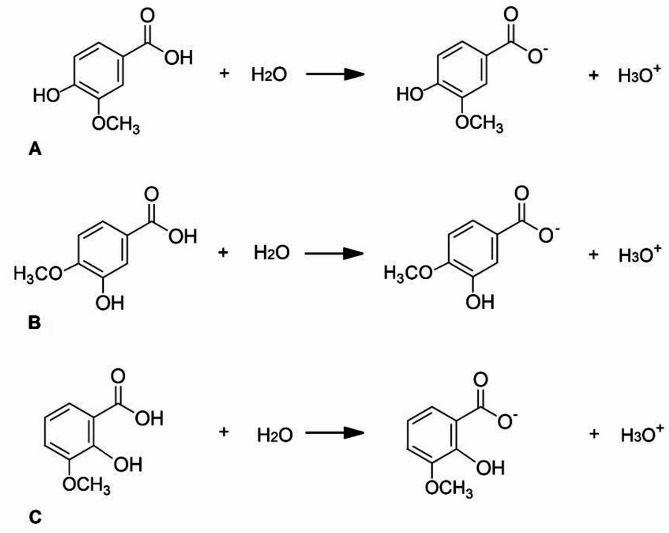
Dissociation reactions of (**A**) vanillic acid, (**B**) isovanillic acid and (**C**) *o*-vanillic acid.

The dissociation energy of vanillic acids (based on the reactions shown in Fig. 6) and the energy of the frontal orbitals HOMO and LUMO for acid anions were calculated. The CPMC calculation model for the aqueous environment was used. According to the pKa data, the strongest of the three vanillic acids is *o*-VA (pKa = 2.85). IsoVA (pKa = 4.35) and VA (pKa = 4.53) are much weaker acids. The reaction energy of the proton abstraction from the carboxyl group of *o*-VA is much lower than the calculated dissociation reaction energies for VA and isoVA (Table 4).

**Table 4:** Dissociation reaction energies of vanillic, isovanillic and *o*-vanillic acids and the energies of the HOMO and LUMO frontal orbitals of acid anions.

	Energy of neutral molecule [10^3^ kJ/mol]	Water energy [10^3^ kJ/mol]	Energy of anion [10^3^ kJ/mol]	Energy of H_3_O^+^ [10^3^ kJ/mol]	pKa	Dissociation energy kJ/mol	Homo [eV]	Lumo [eV]	Energy GAP [eV]
VA	-1603.552	-200.746	-1602.334	-201.783	4.53	196.98	-9.1588	-5.1574	4.0014
isoVA	-1603.548	-200.746	-1602.334	-201.783	4.35	193.46	-8.9876	-5.3035	3.6841
*o*-VA	-1603.561	-200.746	-1602.371	-201.783	2.85	158.13	-9.0715	-5.3819	3.6896

Calculations of the energies of the HOMO and LUMO frontal orbitals and the energy gap ΔE = ELUMO-EHOMO (EGAP) for vanillic acids anions showed that the most reactive anion is isoVA, while the least reactive is VA (Table 5).

**Table 5:** The position of absorption maxima in the electron spectra of vanillic acids.

	Water			Methanol			
	λ_max1_ [nm]	λ_max2_ [nm]	λ_max3_ [nm]	λ_max1_ [nm]	λ_max2_ [nm]	λ_max3_ [nm]	λ_max4_ [nm]
VA	204	254	289	205	218	260	290
*o*-VA	208	-	306	208	-	-	313
isoVA	205	254	290	-	218	258	294

## Experimental study

### IR spectra

**Fig. 7:**
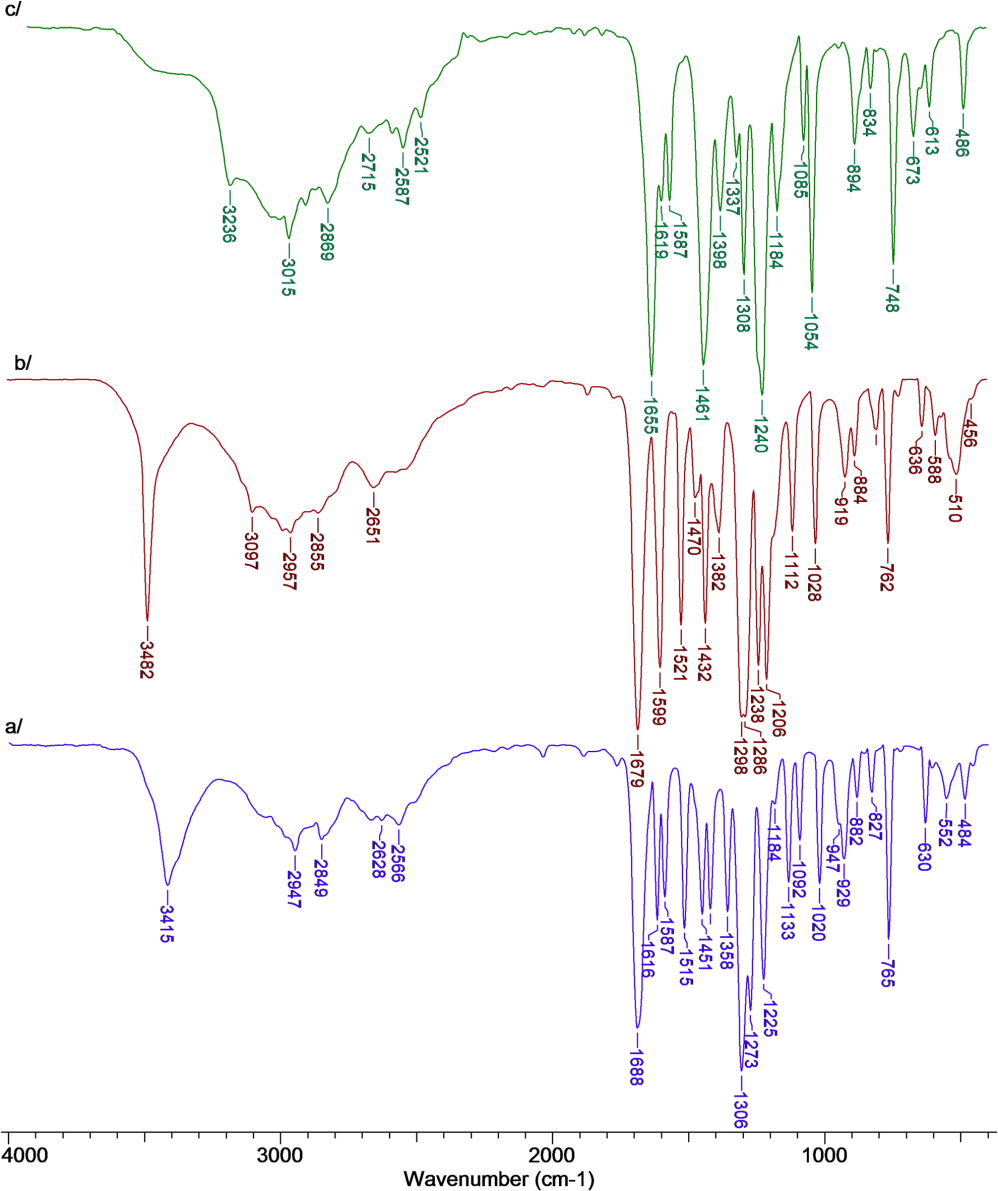
FTIR spectra registered in KBr matrix for (**A**) vanillic acid, (**B**) isovanillic acid and (**C**) *o*-vanillic acid.

The spectra were recorded in the KBr matrix (Fig. 7) and the ATR technique. Table 6 lists the values of the wavenumbers of the bands in the infrared spectra recorded in the KBr matrix, the ATR technique, and the wavenumbers and intensities of the bands in the theoretical spectra calculated for the optimized structures of VA, isoVA and *o*-VA.

**Table 6:** Wavenumbers (cm^−1^), intensities and assignments of bands occurring in the IR (KBr, ATR and DFT) spectra of vanillic, isovanillic and *o*-vanillic acids.

o-VA				VA				IsoVA				Assignment (No.)
IR_KBr_	IR_ATR_	Theor.		IR_KBr_	IR_ATR_	Theor.		IR_KBr_	IR_ATR_	Theor.		
[cm^−1^]	[cm^−1^]	[cm^−1^]	Int.	[cm^−1^]	[cm^−1^]	[cm^−1^]	Int.	[cm^−1^]	[cm^−1^]	[cm^−1^]	Int.	
		3750	166.1			3756	176.8			3755	173.7	
3236 m	3223 w	3642	552.9	3482 s	3479 m	3802	191.9	3415 m	3415 w	3812	150.0	ν(OH)_ar/H2O_
3080 w		3210	7.4	3097 w	3093 vw	3215	1.1	3053 w		3210	2.9	νCC (2)
3015 m	3015 w	3196	5.4	3024 sh		3175	10.6	2978 sh		3191	4.2	νCC (20a)
		3138	33.2			3139	30.9			3145	25.5	ν_as_CH (CH_3_)
2952 m	2952 w	3096	40.6	2957 m	2957 w	3098	40.2	2947 m	2944 w	3109	36.0	ν_as_CH (CH_3_)
2869 m	2869 w	3024	78.7	2855 w	2843 vw	3025	76.2	2849 w	2836 w	3030	83.6	ν_s_CH (CH_3_)
2715 − 2521				2794 − 2537				2666 − 2504				ν(OH)
1655 vs.	1650 s	1745	903.6	1679 vs.	1677 vs.	1726	819.5	1688 vs.	1681 s	1733	805.7	ν(C = O)
1619 m	1617 w	1640	154.4			1632	266.8	1616 m	1616 m	1635	107.8	νCC (8b)
1587 m	1586 m	1615	10.3	1599 s	1597 s	1621	18.0	1587 m	1587 m	1616	126.0	νCC (8a)
		1515	86.7	1521 s	1521 s	1536	162.7	1516 m	1515 s	1545	109.7	νCC (19b)
1461 vs.	1453 s	1494	23.4	1470 w	1464 sh	1494	13.6	1451 m	1453 m	1497	20.8	δ_as_CH_3_
					1456 m			1420 m	1420 m			β(OH)
		1467	132.1	1432 s	1433 s	1447	57.8			1442	110.1	νCC (19a)
1398 m	1396 m	1479	11.8	1382 m	1395 m	1480	15.9	1358 m	1357 m	1479	11.7	δ_as_CH_3_
1308 s	1308 m	1470	133.2		1338 w	1477	30.6			1469	7.5	δ_s_CH_3_
1240 vs.	1236 vs.	1313	136.1	1298 vs.	1300 sh	1353	303.9	1306 vs.	1304 vs.	1362	229.1	ν(C-OH)
		1256	744.0	1283 vs.	1281 vs.	1307	528.8	1273 s	1270 s	1359	126.7	νCC (14)
1184 m	1184 m	1249	8.1	1238 s	1238 vs.	1251	276.2			1253	186.5	νCH (13)
				1206 s	1205 s			1225 s	1226 s			β(OH)
		1249	8.1	1184 sh		1251	276.2	1184 w	1185 w	1253	186.5	ν(O-CH_3_)
1085 m	1083 w											βCH (18a)
1054 s	1052 s	1178	0.7	1112 m	1112 m	1188	26.0	1133 m	1133 m	1177	194.1	βCH (18b)
		1103	57.9		1058 w	1169	144.7	1092 m	1092 m	1173	395.6	βOH; βCH
		1163	9.0	1028 m	1027 m	1165	18.6	1020 m	1020 m	1165	39.2	δ_as_CH_3_
894 m	892 m			919 m	916 m			929 m	926 m			γOH
834 w	834 w	847	34.8	884 w	884 m	910	34.6	882 w	881 m	948	40.0	νCH (7b)
813 vw	813 vw	827	4.5			840	20.2	826 w	828 m	855	9.0	γCH (10a)
748 s	746 s	762	106.0	762 m	760 s	776	92.9	765 s	763 vs.	782	66.3	β(C = O)
		696	51.7	724 w	725 w	722	35.0	722 w	728 vw	762	34.1	αCCC (1)
673 m	667 m			636 w	637 m			630 m	630 m			γ(C = O)
613 w	617 w	614	27.9			620	26.4	604 vw		629	79.2	αCCC (6b)
		595	13.9	588 w		587	40.2	552 w		598	26.2	γ(OH); γCH
564 vw		579	18.4	565 vw		526	82.2			539	100.0	ϕCC (16a)
				537 sh		544	40.3			523	27.0	αCCC (6a)
486 m		494	156.4	510 m		492	11.7	484 w		491	18.1	ϕCC (16b)

s – strong; m – medium; w – weak; v – very; sh – shoulder.

The symbol “ν” denotes stretching vibrations, “β” denotes in-plane bending modes, “γ” designates out-of-plane bending modes; “α(CCC)” denotes the aromatic ring out-of-plane bending modes, and “φ(CCC)” designates the aromatic ring in-plane bending modes.

The bands originating from the vibrations of the carboxyl group in the spectra of VA and isoVA are located at similar wavenumber values. The band of stretching vibrations νC = O in the spectrum of VA is located at 1679 cm^−1^ (KBr), 1677 cm^−1^ (ATR), and in the spectrum of isoVA at 1688 cm^−1^ (KBr) and 1681 cm^−1^ (ATR). The same band in the spectrum of *o*-VA is located at 1655 cm^−1^ (KBr) and 1650 cm^−1^ (ATR). Therefore, a significant shift of the band in the spectrum is observed in *o*-VA compared to the position of this band in the other two acids. The shift of this band in the spectrum of *o*-VA is related to the interaction between the hydroxyl group and the carboxyl group of the acid (the presence of a hydrogen bond). A shift of the ν(C-OH) vibration band in *o*-VA was also observed, compared to the spectra of the other two acids. The position of the aromatic system bands in the three acids studied was analyzed. It is known that the shifts of the aromatic system bands towards lower wavenumbers and the decrease in the intensity of these bands or their disappearance indicate an increase in the destabilization of the electronic system in the aromatic ring. The shifts of the aromatic system bands in the spectra of the three acids studied were compared. It was observed that in the spectrum of *o-*VA the bands originating from the vibrations of the aromatic system are located at lower wavenumber values ​​or the bands are marked in relation to these bands in the spectra of VA. These bands include the bands marked according to the Versany’i numbering: 8a, 19b, 19a, 14, 18b, 7b, 10a, 6b, 16a. Changes in the position of the bands of the aromatic system in the spectra of the acids studied indicate that the electronic system of *o*-VA is more disturbed and less stable than the π-electron system of the other two acids. The influence of the hydroxyl group of *o*-VA on the distribution of the electronic charge in the carboxyl group of this acid and on the distribution of the electron charge in the aromatic system is observed, which affects the reactivity of this acid. Different positions of the methoxy group substituted in different positions in the aromatic ring also affect the electronic system of the aromatic ring, which is reflected in the position of the vibration band of this group in the spectra. The positions of the bands originating from the vibrations of the methoxy group are similarly situated in the spectra of VA and isoVA. However, deviations are observed in the spectrum of *o*-VA. Analysis of the spectroscopic spectra in the infrared (KBr and ATR) confirms the previous observations that the electronic system and the resulting reactivity for VA and isoVA will be at a similar level. On the other hand, *o*-VA is characterized by reduced aromaticity and increased disturbance in the distribution of the electronic charge, so it will be a more reactive system.

### UV-VIS study

**Fig. 8:**
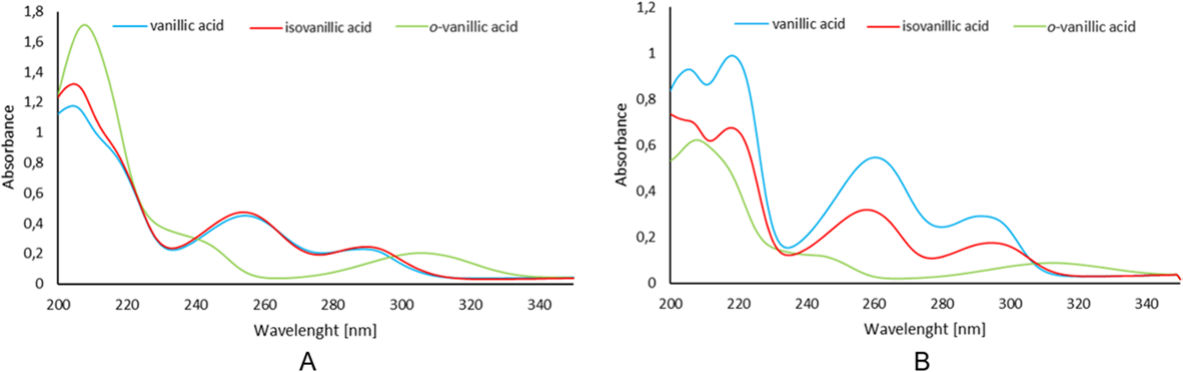
Electronic spectra (200–340 nm) for vanillic acid registered in (**A**) water solution and (**B**) methanolic solution.

Figure 8A shows the absorption spectra of vanillic acids recorded in aqueous solutions, and Fig. 8B presented those recorded in methanol. The bands located in the ranges 204–260 nm are associated with electronic transitions π→π* in the aromatic ring of the acids. The first maximum in the recorded spectrum of VA, designated as λ_max1_, is located at a wavelength of 204 nm (water) and 205 nm (methanol). The positional change of the hydroxyl and methoxy groups in the aromatic ring causes a bathochromic shift of the band. This band is located in *o*-VA at 208 nm (water and methanol) and in isoVA at 205 nm (water). The λ_max2_ band in the spectra of VA and isoVA recorded in methanol is located at 218 nm. This band was not observed in the spectrum of *o*-VA. In aqueous solutions these bands are absent. The λ_max3_ band associated with the electronic transition π→π* in the spectrum of VA and isoVA is located at a wavelength of 254 nm. In the spectra recorded in methanol solutions this band is located at 260 nm (VA) and 258 nm (isoVA). In the spectrum of *o*-VA this band was not observed.

Bathochromic and hypsochromic shifts of the aromatic system bands in the spectra are related to changes in the electronic charge distribution in the aromatic ring. The disappearance of the π-electron system bands indicates an increase in the disturbance of the electronic charge distribution in *o*-vanillic acid compared to the other two acids. The absorption bands marked as λ_max4_ present in the spectra of the acids studied are related to the n→π* electron transition within the carbonyl group C = O. The maximum absorption associated with this electronic transition in VA and isoVA is located at similar wavelengths – 289 nm and 290 nm (in the spectra recorded for aqueous solutions) and 290 nm and 294 nm (in the spectra recorded for methanol solutions), respectively. This band undergoes a bathochromic shift in the spectra of *o*-VA to 306 and 313 nm, respectively, which is caused by the presence of an intramolecular hydrogen bond between the hydroxyl group of the aromatic ring and the carbonyl group^21^.

### Antioxidant properties

**Fig. 9:**
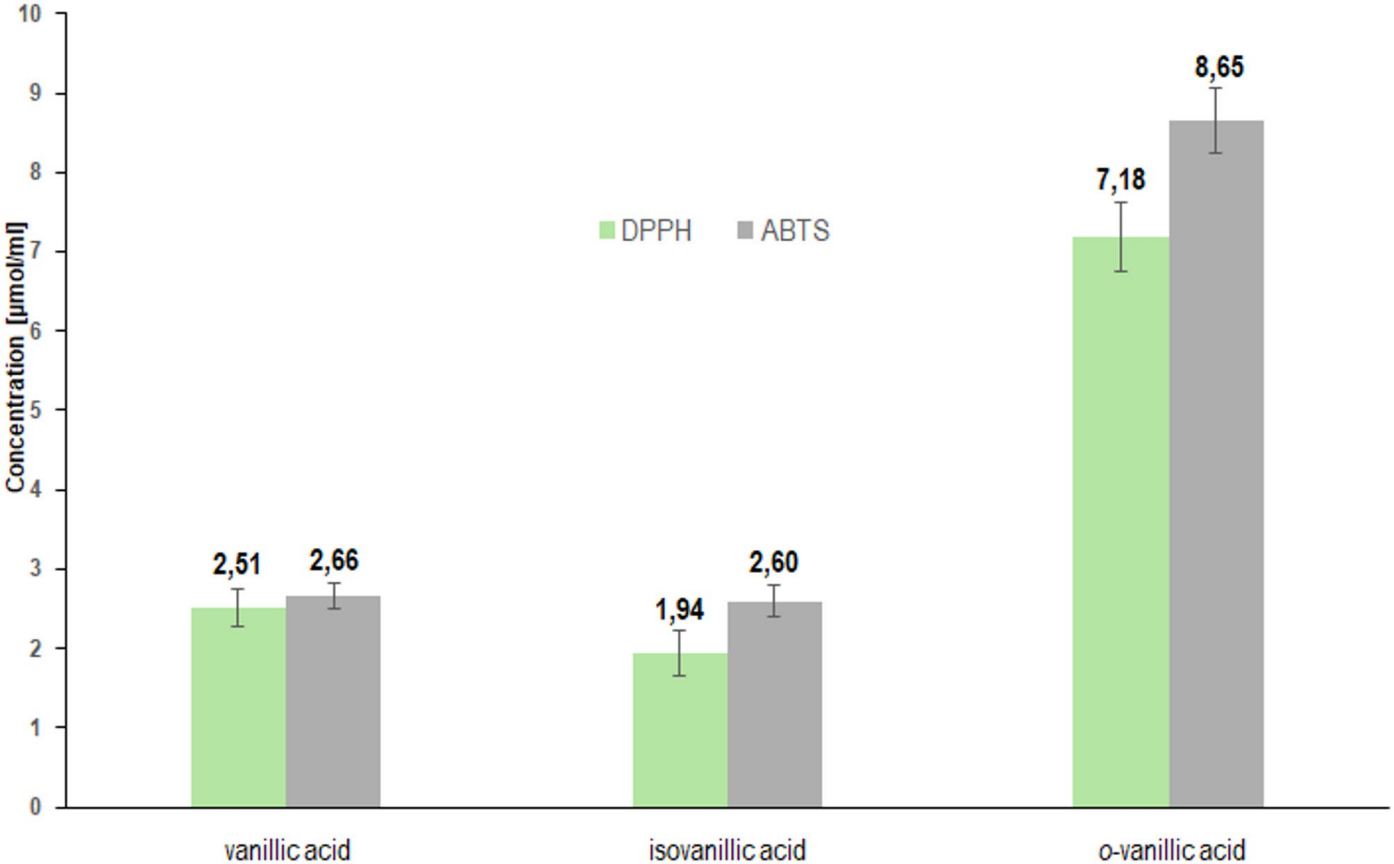
Antioxidant properties of vanillic, isovanillic and *o*-vanillic acids tested with DPPH and ABTS tests (IC50).

**Fig. 10:**
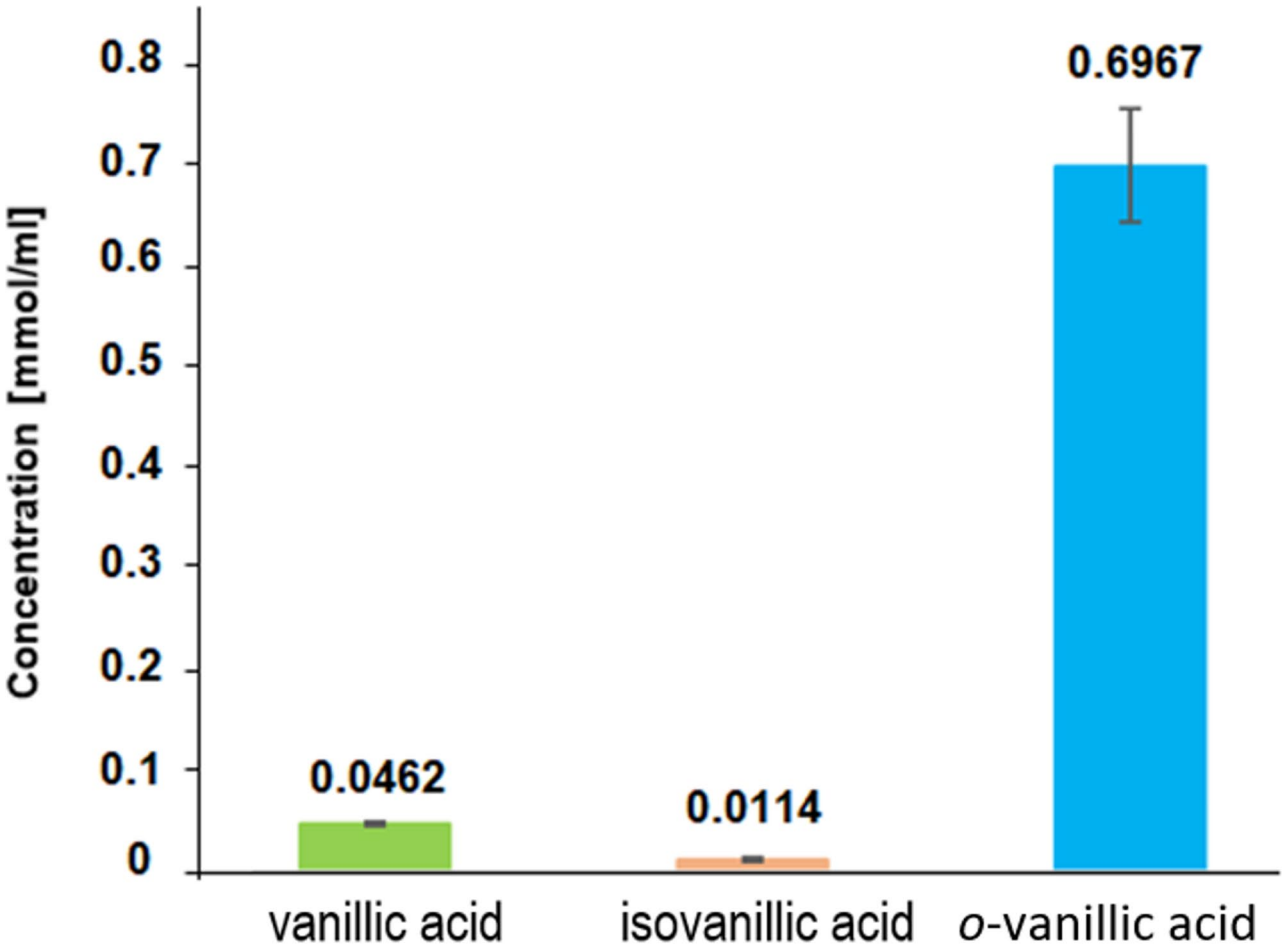
Antioxidant properties of vanillic, isovanillic and *o*-vanillic acids tested with OH radical inhibition test (IC50).

The antioxidant properties of vanillic acids were determined in assays with the DPPH, ABTS and hydroxyl radical HO^•^. The results of the DPPH and ABTS tests are presented in Fig. 9 and the results of the HO^•^ radical test are presented in Fig. 10. The determined IC50 values ​​showed that isoVA exhibits the highest antioxidant potential (IC50 = 1.94 +/-0.20 µmol/l in the DPPH test, 2.60+/-0.19 µmol/l in the ABTS test and 0.011+/-0.00148 mol/l in the HO^•^ radical test). VA has a similar antioxidant potential (2.51+/-0.23 µmol/l, 2.66+/-0.16 µmol/l and 0.046+/-0.00135 mol/l, respectively). *O*-VA acid is the least effective in removing free radicals (7.18+/-0.44 µmol/l, 8.65+/-0.41 µmol/l and 0.697+/-0.05591 mol/l, respectively). For DPPH and ABTS assays, statistical analysis showed no significant difference between VA and isoVA, but *o*-VA differed significantly from both of them. In the case of HO^•^ radical inhibition test the difference between groups VA and isoVA was statistically significant in a pairwise comparison (*p* = 0.0000162); however, it did not remain significant after Tukey’s correction for multiple comparisons (*p* = 0.56). *O*-VA differed significantly from both VA and isoVA. One of the reaction mechanisms of the tested compounds with the DPPH radical is the transfer of a hydrogen atom (HAT), while in the case of the test with the ABTS radical, an electron is transferred from the ABTS cation radical to the hydroxyl group of the antioxidant. In the case of *o*-VA, the proton of the hydroxyl group of the aromatic ring, which participates in the processes of quenching radicals, is located in close proximity to the carboxyl group. Proton dissociation can be significantly influenced by intermolecular and intramolecular hydrogen bonds. Therefore, a change in the concentration of an antioxidant implies the properties of this antioxidant, in extreme cases causing radically different behavior of this oxidant. Antioxidant properties of the same compound can change into prooxidant properties, and vice versa. In our opinion, such different behavior of one compound depends on the formation or decomposition of intermolecular hydrogen bonds of the antioxidant with the solvent. Diffraction studies^12^ have shown that this proton interacts with the oxygen of the carbonyl group (intramolecular hydrogen bonds), which is why the antioxidant potential of *o*-VA is much lower than that of VA and isoVA.

### Cytotoxicity

**Fig. 11:**
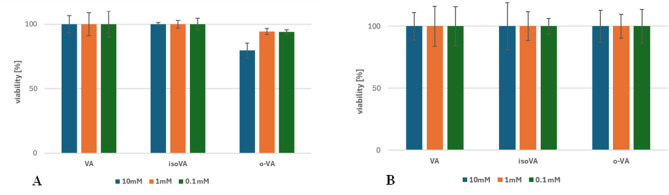
Viability (%) of HEP-G2 (**A**) and Caco-2 (**B**) cells depending on the concentration of the tested vanillic acids: VA-vanillic acid, isoVA - isovanillic acid, *o*-VA- *o*-vanillic acid.

Among the tested compounds, only *o*-vanillic acid showed cytotoxic activity only on the HEP-G2 cell line (Fig. 11). Isovanillic acid did not show cytotoxic properties at any of the tested concentrations on any of the cultured cell lines. Statistical analysis showed no significant difference between activity of VA and isoVa on the HEP-G2 cell line, however, *o*-VA showed a statistically significant difference compared to both (considering all tested concentrations). There was also no significant difference between activity of tested compounds on Caco-2 cell line.

The results of the conducted studies show a complete lack of correlation between antioxidant activity and cytotoxicity toward cancer cells. Compounds with the highest ability to eliminate free radicals (according to DPPH and ABTS tests) showed a complete absence of cytopathic effect on the tested cancer cell lines. However, the only compound (*o*-vanillic acid) that showed 20% cytotoxic activity at the highest tested concentration (10mM) towards the HEP-G2 cell line is a weak antioxidant according to the conducted studies. This confirms the results of the studies by Osman et al.^24^ that the content of phenols may indicate antioxidant scavenging activity, but not cytotoxic activity. In their publication, they showed that the Pearson correlation coefficient between antioxidant activity in relation to the total content of phenols was weakly correlated with cytotoxicity towards liver and breast cancer cells. The decrease in HEP-G2 cell viability demonstrated in our studies by the presence of *o*-vanillic acid may result from anti-cancer mechanism distinct from scavenging free radicals, but for example by inhibiting proliferation, inducing apoptosis or autophagy. It is advisable to extend the studies with in vitro and in vivo biological tests in order to determine which anti-cancer mechanism is the cause of the cytotoxic effect proven in our studies for this particular isomer of vanillic acid. The antioxidant activity of vanillic acid and isovanillic acid proven in the DPPH and ABTS tests is not able to induce a cytotoxic effect on the HEP-G2 and Caco-2 cell lines. Nevertheless, scientific data show that different cancer cell lines show different sensitivity to antioxidant compounds. Therefore, considering the obtained results (high antioxidant activity of VA and isoVA), it should be checked whether these compounds will demonstrate the desired cytotoxic effect on other cell lines.

## Discussion

The molecular structure of a compound determines its reactivity, biochemical and antioxidant activity^7,24,25^. The presence and mutual arrangement of functional groups influences the electron density and charge distribution characteristic of a given molecule. This phenomenon is clearly visible in the case of ligands and their isomers, where changing the position of a substituent can lead to distinct differences in the properties they exhibit. In our previous work on the example of vanillic acid and its iso- and ortho- derivatives, we observed differences in their antimicrobial activity. However, these compounds exhibited comparable toxicity, genotoxicity, and induction of oxidative stress^7^. In this work, we focused on deepening the knowledge on the influence of the molecular structure of vanillic acid isomers on antioxidant activity and cytotoxicity. To our knowledge, this work constitutes the first attempt to compare data from theoretical and experimental studies on the structure of vanillic acid and its isomers and its above-mentioned properties. Theoretical calculations showed differences in the aromaticity of the ring and the reactivity of the acid depending on the position of the hydroxy and methoxy groups in relation to the carboxyl group. The *o*-vanillic acid with the OH group in the immediate vicinity of the carboxyl group was characterized by the lowest aromaticity, the highest dispersion of the electronic charge in the ring, and, consequently, the highest susceptibility to reactions. Spectrophotometric analyses showed changes in the position of the bands of the aromatic system in the spectra of the acids tested, confirming the results of the theoretical calculations on the influence of the *o*-VA hydroxyl group on the distribution of the electronic charge in the carboxyl group of this acid and in the aromatic system. The observed, highest dispersion of charge in the case of *o*-vanillic acid, most probably caused by the formation of an intramolecular hydrogen bond between the hydrogen atom of the hydroxyl group and the oxygen atom of the carbonyl group, had an impact on the lower antioxidant activity of this acid compared to the other vanillic acids tested. Electrostatic potential maps modeled for the three vanillic acids molecules showed the effect of shifting the electron cloud from the oxygen atom of the carbonyl group towards the hydroxyl group in the *o*-vanillic acid molecule - compared to the remaining molecules. This affects the ability of the hydroxyl group to exchange proton in reactions with free radicals.

Considering the cytopathic effect of the studied vanillic acids, only *o*-vanillic acid exhibited, although slight, cytotoxicity toward the HEP-G2 cell line, which was not observed in the case of vanillic acid and isovanillic acid. A similar lack of correlation between antioxidant activity and cytotoxicity was reported by Sammar et al.^25^, who assessed cytotoxicity and the ability to neutralize free radicals in 57 plant extracts. The results showed no significant correlation between these two properties. Some extracts with low antioxidant activity showed strong cytotoxic effects, suggesting that other mechanisms, such as interactions with target proteins or modulation of signaling pathways, may be responsible for their anticancer activity. Similarly to our studies, the lack of cytotoxic effect of vanillic acid on HTC cell lines in the concentration range from 1 to 100 µM was observed in the studies of Almeida et al.^26^. In contrast, in the study of Surya et al.^27^, vanillic acid showed a significant ability to inhibit the proliferation of MCF-7 breast cancer cells, resulting from its ability to induce excessive ROS generation and its potential to induce apoptosis. In the case of studies on the cytotoxicity of vanillic acid derivatives, the number of reports is limited. The above-mentioned work of Matejczyk et al.^7^ reveals, that *o*-vanillic acid showed the highest toxicity and protein degradation potential in *E. coli grpE: luxCDABE* cells after 24 h at a concentration of 100 mg/l, although there were no significant differences in toxicity between isoVA and *o*-VA. The fact that vanillic acid derivative showed cytotoxicity only towards HEP-G2 cells may be the result of a greater sensitivity of these cells to the tested substances than Caco-2 cells. The HEP-G2 and Caco-2 cell culture models are widely used in vitro models for studying the biological activity of natural phenolics. The human intestinal cell line Caco-2 is often used for screening bioaccessibility and cellular uptake of polyphenols^28,29^. Whereas human hepatic HEP-G2 liver cell model is used for drug metabolism and hepatotoxicity^30,31^. Functional differences between the two models, resulting from their distinct tissue origin, may lead to different responses to the tested compounds. Achour et al.^32^ showed that structural differences between the tested phenolic acids, i.e. caffeic, rosmarinic and ferulic acids, caused differences in the type of metabolites formed in the Caco-2 and HEP-G2 cell models, with the metabolic rate being higher after incubation with HEP-G2 than Caco-2 cells. Sahu et al.^33^ in their publication describe that after exposure to 20-nm silver nanoparticles, HEP-G2 showed a significant decrease in mitochondrial membrane potential in a wider range of concentrations than Caco-2. At the same time, no significant oxidative stress was observed in either cell lines may involve a distinct mechanism or may be different in nature. The collected literature data indicates that the mechanism of cytotoxic action of phenolic acids may be multifactorial and depends on the structure of the specific phenolic acid and the type of cells. Our results suggest that the ability of the compounds to scavenge free radicals is not correlated with the observed lack of cytotoxicity against HEP-G2 and Caco-2 cells and that a different mechanism of mitochondrial damage leads to cytotoxicity in the case of *o*-vanillic acid. As demonstrated by Kampa et al.^34^, the antioxidant activity of 3,4-dihydroxyphenylacetic acid (PAA) and caffeic acid in T47D cells does not coincide with their inhibitory effect on tumor proliferation. PAA induced inhibition of nitric oxide synthase, while caffeic acid competed for binding and caused inhibition of the CYP1A1 enzyme induced by the aryl hydrocarbon receptor. Both agents induced apoptosis via the Fas/FasL system. Gong. et al.^35^ in their publication prove that vanillic acid, reduces the proliferation of HCT116 colon cancer cells by arresting the G1 phase of the cell. Moreover, most studies indicate that the cytotoxicity of phenolic compounds is dose- and time-dependent and that, depending on the concentration and conditions, a given compound may exhibit anti- or pro-oxidant activity^36,37^.

Taking into account the current literature data and the results presented in this paper, it is obvious that further research is necessary to determine the mechanism of the cytotoxic activity of vanillic acid and its derivatives towards HEP-G2 cells and towards other cell lines.

## Conclusion

In summary, the studies have shown that modulation of the molecular structure of vanillic acid by changing the mutual position of functional groups influences its electronic structure and reactivity and the resulting differences in antioxidant and cytotoxic activity. *O*-vanillic acid, characterized by the highest reactivity, turned out to be a weak antioxidant, and at the same time, the most potent cytotoxic compound. Theoretical calculations and FTIR and UV infrared spectroscopic studies showed that isovanillic and vanillic acids exhibit greater stabilization of the π electron system and lower reactivity than *o*-vanillic acid. The antioxidant activity of the acids tested is suggested to be associated with the presence of the hydroxyl group and the interactions of its hydrogen atom with the oxygen atom of the carboxyl group. Moreover, the results of the studies indicate a lack of direct correlation between antioxidant activity and cytotoxicity towards cancer cells. Compounds with the highest ability to eliminate free radicals (according to DPPH and ABTS tests) did not show any cytotoxic effect on the cancer cell lines tested. This phenomenon may result from the different nature of the activities considered and differences in the mode of action of the compounds tested. Therefore, due to the observed complexity of the effect of the structure of compounds on the studied phenomena, more in-depth research is necessary to obtain reliable information on the actual activity of vanillic acid derivatives in the human body and their future use in the prevention of lifestyle diseases.

## Materials and methods

### Theoretical calculations

Geometric optimization of vanillic (VA), isovanillic (isoVA) and *o*-vanillic acid (*o*-VA) molecules and radicals and anions was performed using the DFT method (B3LYP/6-311 + + G(d, p)^38^ using water as a solvent (CPCM computational model). The electronic charge distribution was calculated for the optimized structures using the NBO (Natural Bond Orbital)^39^ method. The infrared vibration frequencies and the energy of HOMO and LUMO orbitals were calculated using the (B3LYP/6-311 + + G(d, p) method. Electrostatic potential maps were modeled for geometrically optimized structures of molecules and radicals using the SCF (Self-Consistent Field) method. The energy of the radical formation reaction and the dissociation energy were calculated based on the calculated energies for optimized neutral molecules, radicals and anions. All calculations were performed using the Gaussian 09 program^40^.

Based on the geometric data (calculated bond lengths in the aromatic ring), the aromaticity indices were calculated. Aromaticity of the system π-electron is related to the stability of the electronic system in the aromatic ring. It allows predicting the reactivity of the molecules being studied. Rings with high aromaticity are stable systems with low reactivity and low susceptibility to substitution.

The HOMA (Harmonic Oscillator Model of Aromaticity)^41^ index describes the aromaticity of a system based on the alignment of bonds in the π-electron system. For a “pure aromatic” compound structure with aligned and optimal bond lengths, the aromaticity takes the value of 1, while for a non-aromatic structure, the value is 0. The equation used to calculate aromaticity takes the form:

$$\:\text{H}\text{O}\text{M}\text{A}=1-\left[{\upalpha\:}{\left({\text{R}}_{\text{o}\text{p}\text{t}}-{\text{R}}_{\text{a}\text{v}}\right)}^{2}+\frac{{\upalpha\:}}{\text{n}}{\sum\:\left({\text{R}}_{\text{a}\text{v}}-{\text{R}}_{\text{i}}\right)}^{2}\right]=1-\text{E}\text{N}-\text{G}\text{E}\text{O}$$,

where: R_av_ – average bond length, n – number of bonds considered, R_opt_ – optimal binding length, R_i_ – length of the i-th bond, α – normalisation coefficient.

The Julg’s index (Aj) is calculated based on the equation^42^:

$$\:{\text{A}}_{\text{j}}=1-\left(\frac{225}{\text{n}}\right){{\sum\:}_{\text{r}=1}^{\text{n}}\left\{1-\left(\frac{{\text{R}}_{\text{r}}}{\text{R}}\right)\right\}}^{2},$$

where: *n* – number of CC bonds in the π-electron system, *R*_*r*_ – current CC bond lengths, *R* – average value of the length of all CC bonds.

The BAC (Bond Alternation Coefficient)^43^ index is calculated from the equation:

$$\:\text{B}\text{A}\text{C}=1-\text{3,46}\sqrt{{\sum\:}_{\text{n}}{\left({\text{R}}_{\text{n}}-{\text{R}}_{\text{n}+1}\right)}^{2}}$$

where: R_n_ – length of the nth bond, R_*n*+1_– length of the bond adjacent to the nth.

The aromaticity indices Aj and BAC determine the aromaticity of a given system on a scale of 0 to 1 (non-aromatic system and purely aromatic system). The Bird index is described by equations that take into account the relationship between the order of bond and its length. The aromaticity of the studied systems is determined by a measure on a scale of 100 to 0. Determining aromaticity using the Bird^44^ model requires the use of the following equations:

$${\text{I}}=100\left[ {1 - \left( {{\text{V}}/{{\text{V}}_{\text{k}}}} \right)} \right],$$

where: Vk is 35 for five-membered rings, and six-membered ones 33.3.

V – can be calculated from the equation:

$$\:\text{V}=\left(100/{\text{n}}_{\text{a}\text{v}}\right){\left[{\sum\:}_{\text{r}=1}^{\text{n}}{\left({\text{n}}_{\text{r}}-{\text{n}}_{\text{a}\text{v}}\right)}^{2}/\text{n}\right]}^{\frac{1}{2}}$$,

where: n_av_ – average bond order, n – number of bonds, n_r_ – bond order.

### IR spectra

Infrared spectra were recorded in the range of 4000 –400 cm^−1^, at a resolution of 1 cm^−1^. Transmission spectra were recorded for pellets of the pressed sample with potassium bromide (KBr), in a mass ratio of 1:200 (sample: KBr). Reflectance spectra were recorded using the ATR technique. The spectra were recorded on an Alfa apparatus from Bruker.

**UV-VIS**.

UV-VIS spectra were recorded for methanolic and aqueous solutions at a concentration of 5*10^−5^ mol/dm^3^ using a resolution of 1 nm. The spectra were recorded on a UV-VIS-NIR Cary Agilent Spectrometer.

### Antioxidant studies

#### DPPH^•^

The determination of the antiradical activity of the compounds was performed by conducting a direct reaction of the DPPH^•^ radical, according to the method described in^45^. The control samples contained methanol instead of the tested compound solution. The absorbance was measured at λ = 516 nm with reference to methanol. The result was the DPPH^•^ radical inhibition (%I) calculated using the formula:

$$\:\%I=\frac{{A}_{c}-{A}_{t}}{{A}_{c}}\times\:100\text{\%}$$

Where: A_c_ - absorbance of the control sample, A_t_ - absorbance of the tested sample.

The radical scavenging activity was expressed as the IC_50_ parameter (concentration needed to reduce the initial radical concentration by 50%).

The experiment was carried out in three independent series of five repetitions, and the arithmetic mean was drawn and the standard deviation calculated. The results are shown in the graph.

#### Hydroxyl radical (HO^•^)

The hydroxyl radical inhibition activity was measured according to the method described in^46^. The control samples contained water instead of H_2_O_2_, and the blank samples contained DMSO instead of the tested compound solution. The absorbance was measured at λ = 510 nm, with reference to water. The level of hydroxyl radical inhibition (%I) was calculated using the formula:

$$\:\%I=(1-\left(\frac{{A}_{t}-{A}_{c}}{{A}_{b}}\right)) \cdot 100\text{\%}$$

Where: A_t_ - the absorbance of the tested sample, A_c_ - the absorbance of the control sample, A_b_ - the absorbance of the blank sample.

The radical scavenging activity was expressed as the IC_50_ parameter (concentration needed to reduce the initial radical concentration by 50%).

The experiment was carried out in three independent series of five repetitions, and the arithmetic mean was drawn and the standard deviation calculated. The results are shown in the graph.

#### ABTS^•+^

The determination of the antiradical activity of the compounds was performed by conducting a reaction of the ABTS^•+^ radical, according to the method described in^47^. The absorbance was measured at λ = 734 nm against methanol. The control samples contained methanol instead of the tested compound solution. The result was the ABTS^•+^ radical inhibition (%I) calculated using the formula:

$$\:\%I=\frac{{A}_{c}-{A}_{t}}{{A}_{c}} \cdot 100\text{\%}$$

Where: A_c_ - absorbance of the control sample, A_t_ - absorbance of the tested sample.

The radical scavenging activity was expressed as the IC_50_ parameter (concentration needed to reduce the initial radical concentration by 50%).

The experiment was carried out in three independent series of five repetitions, and the arithmetic mean was drawn and the standard deviation calculated. The results are shown in the graph.

### Cytotoxicity

In this study, complementary biological research methods were used. An in-vitro cytotoxicity test against human Caco-2 colorectal adenocarcinoma cells and HEP-G2 hepatocellular carcinoma cells was carried out. HEP-G2 cells and Caco-2 were obtained from the American Type Culture Collection (ATCC, Rockville, MD, USA) and culture according to the ATTC protocol. The HEP-G2 cells were cultivated in Minimum Essential Medium (MEM) Eagle medium supplemented with 2 mM L-glutamine, 10% fetal bovine serum, and 1% penicillin/streptomycin. The Caco-2 cells were cultured in a medium of the same composition but lacking glutamine. Cells were incubated at 37 °C in a humidified atmosphere with 5% CO_2_ (standard cultivation conditions). Cells were passaged at 80% confluency with 0.05% trypsin − 0.5 mM EDTA.

A colorimetric method- MTS (3-(4,5-dimethylthiazol-2-yl)-5-(3-carboxymethoxyphenyl)-2-(4-sulfophenyl)-2 H-tetrazolium) assay was applied to determine the influence of phenolic acids on accordingly HG2 and Caco-2 cells proliferation. Cells were seeded in the 96-well plates at 10 000 cells/ml density and incubated for 24 h at 37 °C with 5% CO_2_ to ensure their growth. Thereafter, the culture medium was removed and replaced with a medium containing an experimental compound. The final concentrations were 10 mM, 1 mM, and 0,1 mM. The incubation time was 24 h. Absorbance at 492 nm was recorded after 2 h on the Agilent BioTek synergy H1 (Agilent Technologies ING). A blank experiment detecting compound-free samples was performed in parallel. The experiment was carried out in three independent series of five repetitions, and the arithmetic mean was drawn and the standard deviation calculated. The results are shown in the graph.

### Statistical analysis

Statistical analysis was performed using Microsoft Excel with the Real Statistics Resource Pack add-in. Differences in means between groups were assessed using one-way analysis of variance (ANOVA). When a significant overall effect was found, post-hoc pairwise comparisons were conducted using Tukey’s Honestly Significant Difference (HSD) test. A p-value of less than 0.05 was considered statistically significant.

## Data Availability

All data is available from the corresponding author upon reasonable request.
